# Review of Hybrid Membrane Distillation Systems

**DOI:** 10.3390/membranes14010025

**Published:** 2024-01-18

**Authors:** Heng Zhang, Haizhen Xian

**Affiliations:** School of Power, Energy and Mechanical Engineering, North China Electric Power University, Beijing 102206, China; zh_ncepu_edu@163.com

**Keywords:** membrane distillation, hybrid systems, renewable energy, forward osmosis, reverse osmosis

## Abstract

Membrane distillation (MD) is an attractive separation process that can work with heat sources with low temperature differences and is less sensitive to concentration polarization and membrane fouling than other pressure-driven membrane separation processes, thus allowing it to use low-grade thermal energy, which is helpful to decrease the consumption of energy, treat concentrated solutions, and improve water recovery rate. This paper provides a review of the integration of MD with waste heat and renewable energy, such as solar radiation, salt-gradient solar ponds, and geothermal energy, for desalination. In addition, MD hybrids with pressure-retarded osmosis (PRO), multi-effect distillation (MED), reverse osmosis (RO), crystallization, forward osmosis (FO), and bioreactors to dispose of concentrated solutions are also comprehensively summarized. A critical analysis of the hybrid MD systems will be helpful for the research and development of MD technology and will promote its application. Eventually, a possible research direction for MD is suggested.

## 1. Introduction

The shortage of fresh water is one of the biggest challenges nowadays. It is becoming more and more acute with the development of industry and population expansion [1,2,3]. The overall volume of water reservoirs might be enough to meet the current demand, but unfortunately, saltwater, which we cannot drink or use for watering plants directly, accounts for about 96.54% of water. Only 2.53% is fresh water, and less than 0.36% of the fresh water is directly available to humans. In addition, approximately 70% of fresh water is frozen in glaciers and polar ice caps and about 30% of the total fresh water is found mainly as groundwater [4,5,6]. It is projected that by the year 2025, fresh water demand will exceed supply by 56% due to persistent regional droughts and the shifting of the population to urban coastal cities [7]. Therefore, seawater desalination is believed to be the most promising approach to solve fresh water scarcity [8].

Nowadays, the commonly used desalination technologies can be categorized into three generations [9]. The first-generation thermal-based desalination techniques, mainly including multi-stage flash (MSF) distillation, multiple-effect distillation (MED), and thermal vapor compression (TVC), have been developed over the past 70 years [10]. Among them, MSF is the most widely used thermal process, and is mainly used in the Gulf region of the Middle East, where fossil fuels are abundant [11,12]. As alternatives to the first- generation, second-generation desalination techniques based on membrane operations have been extensively used over the past 50 years. The second generation desalination techniques embrace reverse osmosis (RO), nanofiltration (NF), microfiltration (MF), electrodialysis (ED), and so on [7,13]. Among them, RO is the most commonly used membrane process in the industry [14]. However, RO is an energy-intensive process due to the high salinity of the source and the concentration polarization phenomenon [15,16]. Each year, large RO concentrate discharges leads to a significant loss of water resources and a disposal problem [17].

**Figure 1:**
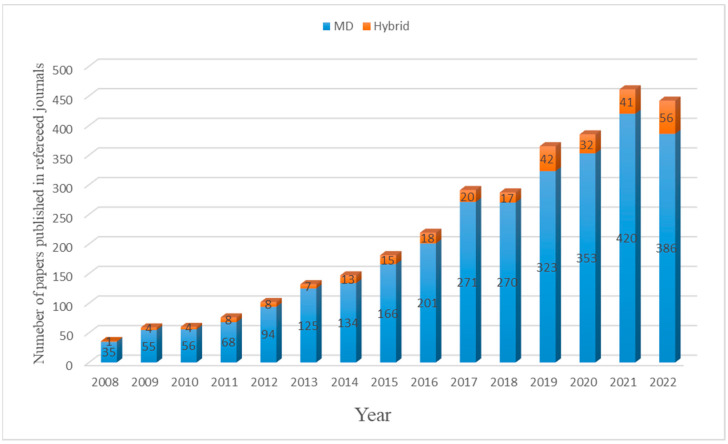
The growth in research activity on MD and hybrid MD, 2008–2022 (articles over the years are sourced from the Web of Science database using the following title search terms “membrane distillation” and ““membrane distillation” AND “hybrid or integrated or coupled or combined””).

Further investigations have been performed to solve the disposal of produced brine and reduce energy consumption. Membrane distillation (MD), forward osmosis (FO), and capacitive deionization (CDI), which can be incorporated into third-generation desalination techniques, have been brought forth as a result [9]. The MD process presents two significant advantages that even RO does not have. On the one hand, the distillation temperature of the MD process is mostly relatively low (currently typ. < 90 °C), which is below the normal boiling point of the feed solution [18,19]. Therefore, it can use waste heat from industrial processes or renewable energy (geothermal energy or solar energy), so it is helpful to decrease the consumption of energy and realize practical/industrial implementations [20]. On the other hand, the membrane of MD is less sensitive to concentration polarization and membrane fouling than other membrane separation processes such as the pressure-driven membrane separation processes [18,21]. Hence, MD can be used to tackle concentrated solutions, especially water recovery from high-salinity solutions, such as RO brine, which is deemed to be the most promising future application of MD by the majority of both academic and industry experts [22]. During the past decade, the number of papers published on hybrid MD has increased significantly (based on the search with titles “membrane distillation” and ““membrane distillation” AND “hybrid or integrated or coupled or combined””) (Figure 1).

Starting from the first patent related to MD (filed in 1963 by Bodell) and the first MD paper (published in 1967 by Findley), there was slow growth in research on MD from the 1970s through the 1990s, but much progress has been made over the past three decades in advancing the development of MD [9,21,22]. The progress and advancements have been reviewed in different review articles. Bourawi et al. [21] comprehensively reviewed the influence of operating variables (feed temperature, feed inlet concentration, feed circulation velocity and stirring rate, permeate inlet temperature, temperature difference and mean temperature effect, permeate flow velocity, and vapor pressure difference) and membrane parameters (such as membrane thickness, membrane porosity, membrane pore size, pore size distribution, pore tortuosity, membrane surface chemistry, and membrane module geometry) on the MD process. Some technical reviews have extensively covered a wide range of commercial MD membranes, membrane synthesis methods used for MD, and recent progress in MD using electrospinning nanofibrous membranes [15,23,24]. The mechanisms of fouling, the control and scaling of fouling, and membrane wetting have also been reviewed in detail [25,26,27]. Hitsov and co-workers [28] provided a critical review of the mathematical modelling and discussed the pros and cons of the different models. Ullah et al. [29] published a comprehensive analysis of the energy efficiency of the DCMD process. However, a few review articles in the literature have discussed the hybrid systems of MD, and some of them are listed in Table 1.

Though these reviews included hybrid systems of MD, they only introduced one aspect of coupling systems. They were not comprehensive. Especially over the past 10 years, a number of studies related to using renewable energy or waste heat to drive the MD process and using MD to tackle concentrated solutions have been undertaken. Thus, it is deemed necessary to provide a comprehensive review of the application of hybrid systems of MD. The structure of this review is outlined below.

The first portion of this review includes a brief overview of the fundamentals and configurations of MD. The second portion of this review emphasizes an up-to-date MD process powered by waste heat and renewable energy sources, such as geothermal energy and solar energy. The third portion of this review focuses on applying MD to treat concentrated solutions from RO, MED, etc. The higher-salinity effluent from the MD process can be used to generate electricity, and can be further treated to achieve zero-liquid discharge (ZLD) or to recover solid crystals such as sodium chloride and valuable minerals.

In the last part, the integration of MD with FO and a bioreactor is described and a future research direction for MD is suggested.

## 2. The Fundamentals and Configurations of MD

### 2.1. Fundamentals of MD

Membrane distillation is a separation technique that couples a thermally driven distillation process and a membrane separation process. This technique has been known for about 50 years [48,49,50,51,52,53]. Simultaneous heat and mass transfer through a micro-porous hydrophobic membrane occurs during the MD process [54]. Heat is transferred by the latent heat carried by water vapor and conduction through the membrane matrix and the vapor trapped inside the pores. With the volatile component being transported through the membrane pores, the mass is transferred [55]. The driving force of the MD process is the partial vapor pressure difference generated by the temperature difference between the hot liquid feed side and the cold permeate side of the membrane or the difference between the water activities of the two solutions [56,57].

### 2.2. MD Configurations

According to types of the driving forces, the configurations of MD can be divided into two categories. The first category includes direct contact membrane distillation (DCMD), air gap membrane distillation (AGMD), sweeping gas membrane distillation (SGMD), vacuum membrane distillation (VMD), and permeate gap membrane distillation (PGMD), the driving forces of which are created by temperature gradients. The other category includes osmotic membrane distillation (OMD), the driving force of which is a concentration gradient.

The most common configuration of MD is DCMD in which hot feed saline water and cold permeate water are in direct contact with the membrane [48]. More than 60% of MD studies related to DCMD systems have been carried out using the simplest configuration of DCMD, whose condensation step can be placed inside the MD module [24]. DCMD requires the least equipment, and its operation is simple [48]. Therefore, it is widely employed for desalination, especially for seawater desalination in remote rural areas where technical support is immature and budgets are small [58]. However, the main drawback of DCMD is the large amounts of heat loss from the hot side to the cold side [59].

The conductive heat loss of DCMD can be significantly reduced by introducing some stagnant air between the membrane and the condensation surface [59]. This configuration is called AGMD, and it has the highest energy efficiency among the single-stage MD modes [60]. In addition, internal heat recovery is more probable for AGMD compared to other modes [61]. Nonetheless, additional resistance to mass transfer is created by the stagnant air when the water vapor passes through the gap to the condensation surface, which results in lower permeate fluxes and is considered a disadvantage [62].

The lower permeate fluxes of AGMD can be avoided if a cold inert gas is used to sweep the vapor on the permeate membrane side to condense outside the membrane module, or if a vacuum pump is applied to the distillate side of the MD module to remove the permeated molecules from the distillate side [59,61]. These configurations are known as SGMD and VMD, respectively. Both of them enhance the mass transfer coefficient and reduce the heat loss due to conduction, and VMD presents the highest permeate flux and lowest heat loss due to conduction among DCMD, AGMD, SGMD, and VMD [54,59,61]. SGMD and VMD are typically used to remove a volatile organic or dissolved gas from an aqueous solution [63]. However, the main disadvantages of SGMD and VMD are that they increase the processing costs due to the additional condenser and vacuum pump, and that they make heat recovery difficult because the condensation takes place outside the membrane module [61].

To solve the decrease in flux caused by the air gap, special materials, such as polyurethane, polypropylene mesh, sand, or de-ionized water, can also be used to replace the stagnant air [64,65]. This configuration is known as material gap membrane distillation (MGMD), which can be understood as a modification of AGMD [66]. In reality, only permeate water can be used in the gap of the module to avoid distillate contamination [65]. Therefore, permeate gap membrane distillation (PGMD) is studied more than polyurethane, polypropylene mesh, and sand gap MD. Simultaneously, the PGMD configuration directly couples the heat recovery within the module better than SGMD and VMD [67].

OMD is a DCMD variant that combines DCMD and OD in one process [68]. The OMD process, which was patented at the end of the last century, is most often used to remove water from liquid foods, such as fruit and milk, and various nonfood aqueous solutions that are not thermally resistant [68]. The main advantages of OMD are that it can concentrate solutions at lower temperatures compared with other MD configurations and effectively increase the transmembrane fluxes by introducing an activity gradient [69,70]. However, OMD is strongly affected by the concentration polarization, particularly on the permeate side [71]. A comparison of the different MD configurations is given in Table 2.

## 3. Renewable Energy and Waste Heat Coupled with MD

**Figure 2:**
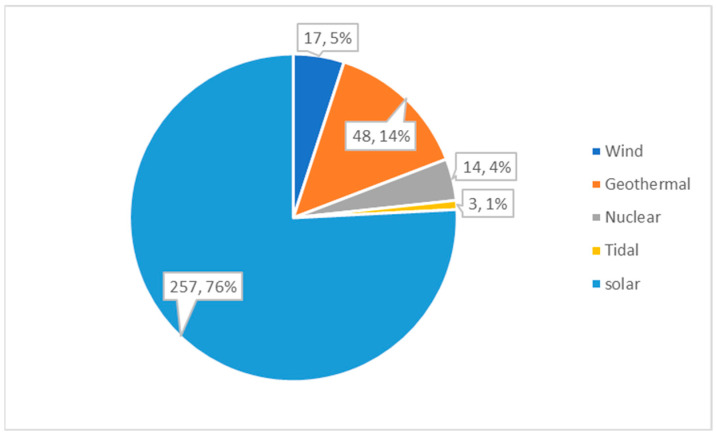
Distribution of investigations related to the MD driven by renewable energy, 2005–2020 (articles over the years are sourced from the Web of Science database using the following abstract search terms ““membrane distillation” AND “solar/wind/tidal/geothermal/nuclear””.

MD is a phase-change desalination process that requires plenty of energy to achieve separation [50]. The use of renewable energy and industrial waste heat in the MD process not only reduces the consumption of conventional fossil fuels, such as oil and gas, but also decreases carbon dioxide emissions and air pollution due to the combustion of fossil fuels [72]. The main conventional renewable energies for desalination include solar and geothermal. A comparison of the number of journal articles from Web of Science about “membrane distillation driven by renewable energy” is given in Figure 2. It shows that solar powered MD processes have been the most widely investigated and implemented, representing 76% of the studies. The second most investigated renewable energy is geothermal, which accounts for 14% of the studies. Wind, nuclear, and tidal energy represent 5%, 4%, and 1% of the published articles, respectively.

Besides the conventional renewable energy sources, industrial waste heat is a source that is already available and has great utilization value. As will be discussed in Section 3.3, waste heat can be acquired from industrial processes and has great potential to power MD processes and lowers the operating cost of water production [73]. In the following subsections, the author presents a detailed introduction to each renewable-driven MD configuration.

### 3.1. Solar Energy-Driven MD

#### 3.1.1. Solar Radiation-Assisted MD

The most abundant and focused renewable energy source is solar radiation, which provides virtually unlimited energy [30,74]. Solar-driven MD desalination processes have been extensively studied by many researchers and practitioners [36]. One of the reasons for interest in solar energy driving MD is that MD has the ability to tolerate discontinuous, fluctuating, and unpredictable operating conditions and to run with a small temperature difference [37,75,76]. Solar radiation can be used to heat a feed solution directly or indirectly via solar collectors, such as flat plate collectors (FPCs), evacuated tube collectors (ETCs), PV panels, and compound parabolic collectors (CPCs), and can also be applied to generate electricity via photovoltaics (PVs) to run auxiliary equipment such as circulation pumps and valves for automatically operated systems [30,36,77].

**Figure 3:**
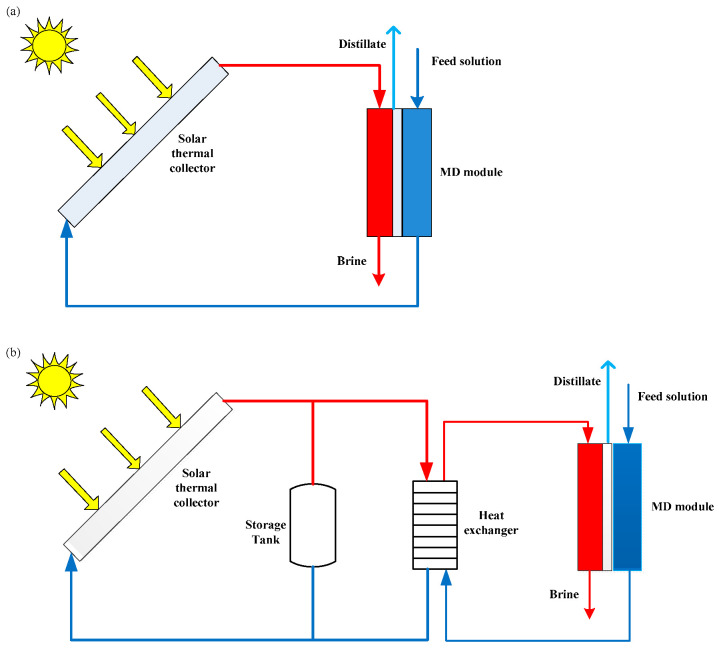
Scheme of solar-driven MD system: (**a**) single-loop system; (**b**) two-loop system.

Two different layouts can be adopted in solar-powered MD desalination systems: single-loop and two-loop systems [78,79]. In a single-loop system (often referred to as a compact system), the seawater is heated directly by solar collectors, and then enters into the membrane module (Figure 3a) [79,80]. The advantages of such systems are that they are suitable for small-capacity production and that the configuration is very simple, without a heat exchanger or heat storage facility [76,81]. However, the materials used in the solar collectors must be anti-corrosion and anti-scaling due to the seawater recirculating through the single loop [36,79]. Compared to a compact system, a two-loop system (Figure 3b) is more complex. This configuration includes two independent loops, a solar loop and a desalination loop, connected by a heat exchanger [36,82,83]. The solar loop is operated with tap water as a heat transfer fluid, while the desalination loop is operated with seawater [77]. Hence, it can achieve better control of corrosion and scaling problems [79]. A two-loop system can also use a heat storage facility to store surplus energy if enough radiation is available, which allows the extended operation of MD modules after sunset [36,79]. However, the maximum feed temperature of the seawater in a desalination loop is lower than the highest temperature in a solar loop due to the energy loss of the heat exchanger [79].

Many experiments and simulations of solar-assisted MD systems for desalination have been carried out by several researchers. Chang et al. [84] have analyzed the performance of a solar membrane distillation desalination system using both experimental and simulation approaches. The authors used a dynamic mathematical model, which included a control algorithm and was verified using experimental results, to optimize the analysis of the system and revealed the operation strategy for maximum water production. The performance of the system was about 80% of the maximum water production for sunny day operation. Zaragoza et al. [85] evaluated various configurations of solar-driven MD and came to the conclusion that spiral-wound modules or multi-effect systems should be considered to improve efficiency. Raluy et al. [86] presented a five-year operational period of a solar-assisted single-loop MD system installed in Spain. They found that the water production ranged between 5 and 120 L/d, and the conductivity of the distillate water varied between 20 and 200 μS/cm. Gil et al. [87] designed four direct control schemes and a reference governor in a solar-powered MD facility. They reached the conclusion that settling times were reduced by more than half and could obtain the operation temperature at the inlet of the distillation module. Gil et al. [88] also proposed a two-layer hierarchical control system that has been tested in a simulation and an experiment to optimize the solar-driven MD facility according to distillate production and thermal energy. The authors concluded that the daily distillate production could improve to 14–20 L and the thermal energy demand could be reduced to 0.41–1.21 kWh/m^3^. Chen et al. [89] proposed a pseudo steady-state method to assess the total annual cost (TAC) of a discontinuous and fluctuating solar-assisted MD desalination facility. They reached the conclusion that the optimal TAC was about USD 280,000 at 500 W/m^2^. Abdallah et al. [90] designed a completely autonomous solar-driven MD unit, in which the feed solution was heated by solar collectors and the electric power of the auxiliary equipment was generated by PV panels. Simulations of the unit operating showed that distillate production varied from 35 L/h to 70 L/h. Additionally, Table 3 summarizes the performances of some solar-assisted MD systems.

Some efforts have also been made to evaluate the economy of solar-driven MD systems. Saffarini et al. [81] found that a DCMD system with a heat recovery device was more cost-efficient than an AGMD or VMD system. Banat and Jwaied [94] calculated the distillate production cost of compact and large autonomous solar-driven MD systems. The costs were USD 15 USD/m^3^ and USD 18 USD/m^3^, respectively, and could be significantly reduced by prolonging the lifetimes of the membranes and units. Moore et al. [95] optimized an SGMD desalination system with solar thermal collectors and PV panels. They found that the cost of water was about USD 85 USD/m^3^, with the membrane modules and thermal collectors accounting for the majority of the cost. In addition, some simulations and theoretical analyses of solar-powered multi-stage MD have been carried out. It was found that it had better performance than a one-stage system in terms of water production, thermal efficiency, and flux [32,58,96,97].

Although the cost of the distillate produced by a solar-driven MD system is relatively expensive compared with other conventional desalination technologies (e.g., RO, MED, and MSF), solar-powered MD systems remain applicable for potable water production in remote and arid areas [34]. In addition, a solar-powered MD system can be energy self-sufficient and requires little maintenance [53]. Solar-powered MD systems are more commonly applied in small applications. Therefore, more studies should be conducted to assess and evaluate the actual operation of large-scale solar-assisted MD units [36]. In addition, more experiments related to solar-assisted multi-stage MD systems are needed to enhance the performance of the systems.

#### 3.1.2. Salt-Gradient Solar Pond-Powered MD

A salt-gradient solar pond (SGSP) is a stable artificial saltwater pond that is used to absorb and store solar radiation [98,99]. An SGSP consists of three characteristic zones: an upper convective zone (UCZ), a non-convective zone (NCZ), and a lower convective zone (LCZ) [100]. The UCZ is at the top of the pond, including a relatively thin layer of water with very low salinity, and the temperature of the water is close to the ambient temperature [101,102]. The NCZ is under the UCZ, and the saline density and temperature of the water in the NCZ increase gradually with depth. Its function is to suppress the convection process in the pond and act as an insulator between the UCZ and the LCZ [101,102]. The last zone is the LCZ, which is at the bottom of the pond. The saturated brine in the LCZ can absorb and store solar radiation to heat buildings or provide a hot feed for the MD process [103,104]. SGSPs have been widely researched due to their low cost and ability to store solar radiation for a long time [105]. Recently, several studies have been conducted to analyze the performance of an SGSP-powered DCMD system. Suárez et al. [106] developed a model to evaluate the performance of an SGSP-driven DCMD system. The authors found that it was possible for distillate production to reach about 2.7 × 10^−3^ m^3^d^−1^ per m^2^ of SGSP. Soon after, Suárez et al. [107] conducted the first experimental study of an SGSP-powered DCMD system to produce fresh water. They reached the conclusion that the average distillate flux of the coupled system could obtain 1.0 Lh^−1^ per m^2^ of membrane. Nakoa et al. [108] carried out an experiment using an SGSP-assisted DCMD system for fresh water production. They found that the fluxes would decrease due to the significant concentration and temperature polarization induced by laminar flow. Suárez and Urtubia [109] investigated the performance of an SGSP-powered DCMD system. The authors reached the conclusion that the maximum fresh water flow rates were about 3.0 L d^−1^ per m^2^ of solar pond.

Membrane distillation powered by a salt-gradient solar pond is an economical and sustainable method for desalination to produce drinkable water. Further research into the economics and practical applications of the systems is needed.

### 3.2. Geothermal Energy-Assisted MD

Compared to solar energy, geothermal energy has the advantage of continuous reliability, availability, stability, and independence from the weather [110,111,112]. In the process of producing drinkable water, geothermal water can directly or indirectly be used as a feed solution to desalinate or for heating feed water from other sources with heat exchangers, respectively [113,114]. However, it is not suitable for MSF due to the low grade of geothermal heat or MED because of the corrosive chemical species brought by geothermal water [30,115]. In a coupled geothermal and RO system, it is necessary to convert geothermal heat into electricity to power a RO plant, which causes huge energy losses [30,116]. MD can make full use of low-grade geothermal water and resists corrosion. Therefore, it can be coupled effectively to geothermal water for desalination. However, it has not been widely studied. Recently, Sarbatly and Chiam [117] presented an energy evaluation of geothermal water acting as a feed solution for VMD to produce drinkable water. The authors reached the conclusion that the cost of water production was USD 0.5 USD/m^3^ for a 20,000 m^3^/d VMD desalination plant operated with geothermal energy, and the cost increased by 144% without using geothermal energy.

Geothermal energy-powered MD desalination is promising. Therefore, more studies should be conducted to address the problems of long-term operation and the corrosion of equipment [115,118].

### 3.3. Waste Heat-Powered MD

Waste heat is everywhere in life, especially in industrial production. MD desalination research has been widely conducted to produce high-quality water using waste heat from industrial production. Four different types of waste heat sources were selected for MD studies, including engine waste heat generated by marine vessels, natural gas compressor stations, a recirculating cooling water system, and a gas-fired power station [73,119,120,121]. In their study, Koo et al. [119] optimized the performance of a VMD process driven by waste heat generated from marine vessels. They analyzed the parameters of the operating conditions and found that an increase in the temperature of the feed water would result in an increase in water flux. Lokare et al. [73] evaluated the potential of a DCMD desalination system powered by waste heat from natural gas compressor stations. They developed a mathematical model to predict the flux performance. They came to the conclusion that the flux and heat recovery efficiency increased with the feed temperature. Waste heat from a recirculating cooling water process was utilized for a DCMD system to produce pure water, which reduced the cost of pure water production and maintenance of a pipeline [120]. Dow et al. [121] explored the viability of DCMD driven by waste heat from a gas-fired power station. The authors found that the system had no significant flux change due to a pretreatment or the unique fouling mechanisms in MD.

Studies show that cheap waste heat can be used by MD systems to produce high- quality distillates. It is financially beneficial to use waste heat to power MD desalination systems due to its competitiveness compared to RO and MED [122]. Therefore, more studies related to waste heat-driven MD systems should be conducted.

## 4. Disposal of Concentrated Brine with MD

**Figure 4:**
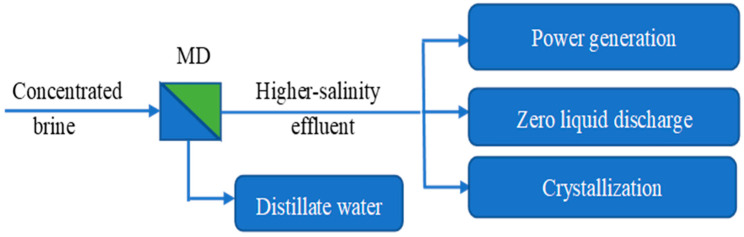
Scheme of an MD hybrid process for disposal of concentrated brine.

It is a big challenge to dispose of the highly concentrated effluent by-products of desalination processes [123]. Discharging the effluent into the ocean in huge amounts would disturb the ecosystem and have negative impacts on the environment [124,125]. Injecting the effluent into the ground would cost a lot and would also pollute fresh water aquifers [126]. Therefore, new ways to dispose of the effluent should be developed for sustainable and cost-effective desalination [127]. MD is a promising way to dispose of concentrated brine due to its low sensitivity to salt concentrations [128]. It can increase the recovery ratio of water whilst minimizing the volume of concentrated brine [129,130]. As shown in Figure 4, higher-salinity effluent can be used to generate electricity by coupling it with the pressure-retarded osmosis (PRO) process, the reverse electrodialysis (RED) process, the capacitive mixing (CAPMIX) process, or further treatment to achieve zero liquid discharge (ZLD) [131,132]. It can also be combined with crystallization to recover solid crystals such as sodium chloride and valuable minerals [133,134].

### 4.1. Power Generation

#### 4.1.1. PRO-MD Hybrid

**Figure 5:**
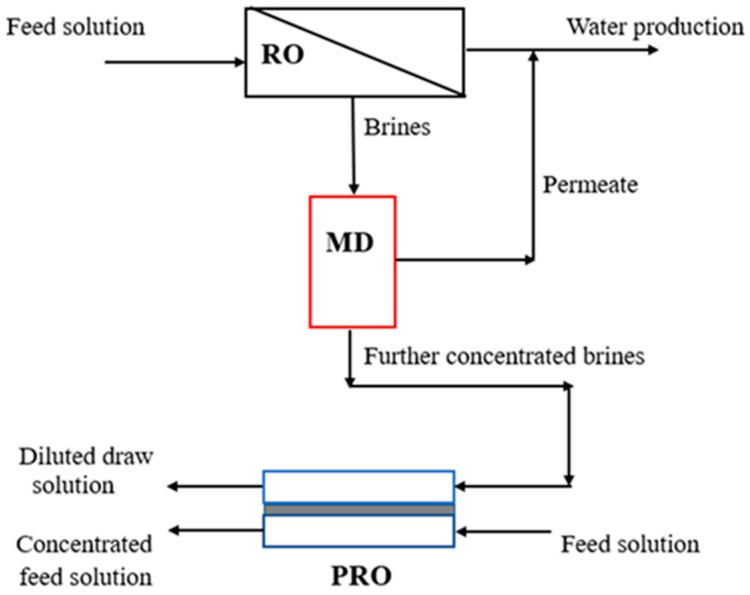
Schematic of RO-MD-PRO hybrid system.

Compared to other desalination processes (such as RO and MED), MD can dispose of a wide range of highly concentrated feed solutions [135,136]. Therefore, MD can act as a complementary process to further concentrate brines from a RO system or a MED system to enhance the recovery of drinkable water and reduce the volume of brines [137,138,139]. Simultaneously, higher-salinity effluent can be supplied to a pressure-retarded osmosis (PRO) system for osmotic power generation [140]. The concept of a RO-MD-PRO system is shown in Figure 5. In the RO-MD-PRO process, MD further concentrates the distillate of RO to generate concentrated and pure water, and PRO converts osmotic energy into electricity using a hydro-turbine, which can save energy [141,142,143]. Kim et al. [144] analyzed the feasibility of the RO-MD-PRO hybrid process using numerical approaches. The authors found that the RO-MD-PRO hybrid process reduced the special energy consumption and minimized marine environmental impacts compared to stand-alone RO process. The maximum SEC was ~1.6 kWh/m^3^ at a brine division ratio of 1.0, which was a 17% reduction compared to the stand-alone RO process. Choi et al. [145] also evaluated the performance and economics of the RO-MD-PRO hybrid system using a theoretical analysis. They obtained results similar to Kim et al. Chae et al. [146] proposed a new dimensionless performance index to compare the energy efficiency between RO-PRO and RO-MD-PRO systems after running several simulations. Using low-grade energy, the simulation results showed that the RO-MD-PRO system had a higher energy efficiency than the RO-PRO system. The simulation results of this research may provide a new roadmap for the study of PRO-hybridized processes. Lee et al. [147] carried out a numerical analysis of a combined multi-stage VMD (MVMD) and PRO system and theoretically studied the distillate and power production of the MVMD-PRO system with different inlet feed flow (from 3 kg/min to 12 kg/min) rates and recycling flow ratios (5–90%). In their research, a maximum power density of 9.7 W/m^2^ was achieved in the case of feed and draw solutions at 0.5 kg/min and a constant hydraulic pressure difference. Several studies related to the performance of the MD-PRO system are summarized in Table 4. From Table 4, we can see that the MD-PRO system can produce both fresh water and electricity. Simultaneously, the energy efficiency of the system is also improved.

Unfortunately, research on the proposed hybrid system has focused on numerical approaches and theoretical analysis, and few studies have experimentally analyzed the hybrid system. Consequently, more experiments should be conducted on the hybrid system to improve its commercial practicability.

#### 4.1.2. MD-RED Hybrid

In the combined membrane distillation and reverse electrodialysis (MD-RED) system, RED converts the Gibbs free energy by mixing the concentrate produced by MD and a diluent, which can convert low-grade heat into electricity [148,149,150]. Several studies related to the hybrid MD-RED system have been conducted by Tufa et al. and Long et al. [131,151]. They reported that a larger NaCl concentration induced greater electrical efficiency. Mercer et al. [150] tested the integration of MD with RED to provide pure water and electrical power and provided the first successful demonstration of a hybrid MD-RED system for energy recovery from waste heat, which was an opportunity for off-grid decentralized sanitation. In another study, Micari et al. [148] provided a detailed description of the behavior of a real RED-MD heat engine and evaluated the performance of the combined system under different operating conditions (such as concentration, velocity, and membrane thickness). Although the integrated MD-RED system outperforms the MD system, the specific costs of the MD and RED equipment are still high, which restricts the development and application of the hybrid system. Therefore, an optimization of the membrane should be performed to enhance the performance of the hybrid process. In addition, a thermo-economic analysis should be conducted for its commercial implementation.

### 4.2. MD-ZLD Hybrid

In recent years, zero-liquid discharge (ZLD) has attracted renewed interest worldwide because it has the advantages of decreasing environmental pollution and improving water sustainability [136,152]. In order to achieve ZLD, thermal-based technologies, such as brine concentrators and solar ponds, and membrane-based ZLD technologies, such as ED, MD, and FO, can be used [136,152]. Tong et al. [152] highlighted the evolution of ZLD from thermal-based to membrane-based processes by thoroughly analyzing the pros and cons of these technologies. Among these technologies, more and more attention has been paid to MD [153]. Schwantes et al. [153] presented a techno-economic comparison of MD and mechanical vapor compression (MVC) in the same ZLD application. They came to the conclusion that MD could be about 40% cheaper than MVC when the distillate was 100 m^3^/day and up to 75% cheaper under the condition of a free waste heat-assisted MD system. Tufa et al. [154] proposed a novel approach integrating DCMD with RED systems to dispose of SWRO brines. The coupled systems could not only produce distillate water via an MD process and generate electricity via a RED process but also achieved near-zero-liquid discharge. Nakoa et al. [155] discussed the combination of a DCMD system with a SGSP to achieve ZLD desalination. The authors used the hot concentrated water in the NCZ as a feed solution and injected the discharged saline water into the LCZ of the SGSP. The integrated systems may lead to ZLD desalination, while the treatment capacity of the concentrated solution is limited.

Regarding the further development of using MD to achieve ZLD, more research on scaled-up MD plants should be conducted, and the membrane lifetime should be extended. ZLD will become more and more attractive and promising.

### 4.3. MD Crystallization

**Figure 6:**
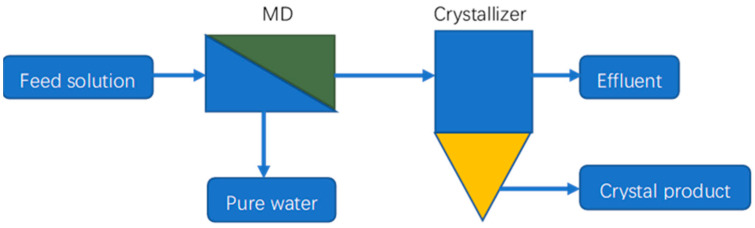
Schematic of an MDC system.

Membrane distillation crystallization (MDC) is an integrated process of MD and crystallization in which pure water is produced via MD and the concentrated brine from the MD system is sent to a crystallizer to recover a valuable crystal product [39,156,157]. A schematic of an MDC system is shown in Figure 6. Edwie and Chung [158] analyzed an MDC system used to treat saturated brine solutions to produce pure water and salt crystals. They came to the conclusion that as the feed temperature increased, the yields of NaCl crystals and pure water would increase, and so would the scaling and membrane wetting. The membrane flux increased first then reduced sharply when the feed temperature was over 60 °C or 70 °C due to the occurrences of membrane scaling and wetting facilitated by salt oversaturation at the boundary layer. Ji et al. [159] conducted an experimental study on the treatment of artificial RO concentrates and natural RO brines using MDC. The authors found that crystallization kinetics were greatly affected by organics in the natural RO retentate. Ali et al. [160] carried out experimental and theoretical research on the treatment of produced water using an integrated MF plus DCMD/membrane crystallization system. By comparing the performances of the integrated processes and MSF in terms of process intensification metrics, the authors reached the conclusion that the coupled system had a better performance than the MSF system according to the productivity/size ratio and productivity/weight ratio metrics.

Further applications of MDC have been carried out in the treatment of industrial wastewater to recover lithium chloride, boric acid, sodium sulfate, and ethylene glycol [161,162,163,164]. MDC also serves as a potential method to realize ZLD. Guo et al. [157] and Chen et al. [165] have explored the use of MDC to achieve ZLD.

However, the potential fouling, scaling, and wetting of the membranes caused by organic materials and inorganic scales are serious problems in the treatment of concentrated brine and will cause reductions in permeate quality, permeate flux, module efficiency, and the lifetimes of the membranes [166,167,168,169,170]. Thus, further studies related to membrane fouling control and scaling reduction are needed before the commercialization of MD systems for the disposal of concentrated brine [166,171,172]. Simultaneously, a long-term stable process of concentrated brine treatment using MD is also needed.

## 5. Other Hybrid MD Systems

In general, coupled MD systems have been widely studied and applied for their ability to tackle brine and use low-grade energy. New types of MD hybrids with mechanical vapor compression (MVC), adsorption desalination (AD), FO, or bioreactors have been researched [173,174,175,176]. Among the new hybrid MD systems for solution treatment, the integration of MD with FO or a bioreactor is most common.

### 5.1. Integration of MD with FO

**Figure 7:**
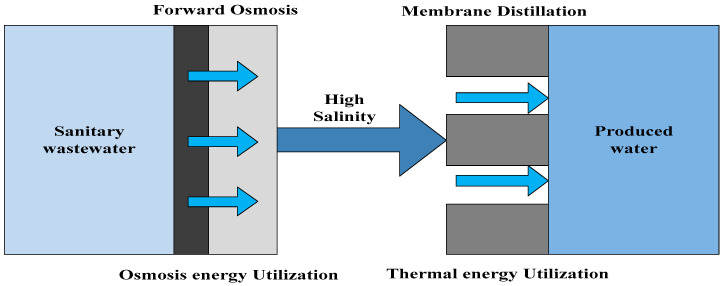
Schematic diagram of lab-scale FO-MD hybrid system.

As a new membrane process, FO has attracted a lot of attention due to its low-fouling characteristics [177,178]. Its driving force is the osmotic pressure difference between the feed solution and the draw solution, which plays an important role in the FO process [179,180]. Some draw solutions that have been applied include NaCl, MgCl2, glucose, high charge of phosphate, EDTA-2Na, and so on [181,182,183]. Wang et al. [173] and Liu et al. [184] have reported that a FO-MD hybrid system could be stabilized at different NaCl concentrations. Nguyen et al. [180] demonstrated that a MgCl2 draw solution with small concentrations of Al2(SO4)3 performs better in water flux compared with pure MgCl2 because of its high osmotic pressure and lower reverse salt flux. However, the FO process must be coupled with other techniques to re-concentrate the draw solution and produce fresh water because it is a dilution process [178,185]. MD is an excellent process to regenerate a FO draw solution compared with conventional pressure-driven membrane processes such as MF, UF, NF, and RO [183,184,186]. As shown in Figure 7, the FO-MD hybrid system is a closed-loop system in which the FO process is used to dewater the feed solution and provide a pretreatment stage for the subsequent MD process, which solves the problem of MD membrane fouling and improves energy efficiency and water recovery, whereas the MD process is employed to the recover the diluted draw solution and produce pure water [178,186,187,188].

Recently, a FO-MD hybrid system has received wide attention for treating high-concentration solutions. Zhou et al. [189] optimized the operating conditions (such as the flow rates of the feed solution and draw solution and the concentration of the draw solution) of the FO process and the temperature of the inlet solution in the MD process. The authors found that the combined FO-MD system that had been optimized could be applied to efficiently treat high-salinity hazardous, and the coupled FO-MD system was more effective than individual FO or MD systems in the rejection of contaminants. Husnain et al. [178] designed an original three-channel FO-MD module for wastewater reuse and Lu et al. [190] applied the three-channel FO-MD module integrated with UF to treat oily water. The three-channel FO-MD module has the advantages of an inherent flux balancing mechanism and a compact structure. However, the heat conduction loss of the module increases. Therefore, further research to improve the energy efficiency of the coupled FO-MD system is needed. In addition, the antifouling ability of the membrane also needs to improve due to the accumulation of contaminants in the draw solution [190,191].

### 5.2. Integration MD with Bioreactor

MDBR is a hybrid system that combines a bioreactor and MD, and can be used to effectively remove trace organic contaminants (TrOCs), such as carbamazepine, oxybenzone, and steroid hormones, and produce volatile materials, such as distillate water and ethanol [174,192,193,194,195,196,197,198]. Wijekoon et al. [197] evaluated the removal of TrOCs by an MDBR system. The authors found that the MDBR system could effectively remove more than 95% of all TrOCs and was not affected by the performance of the bioreactor. However, the removal of total nitrogen and recalcitrant TrOCs was reduced due to the salinity build-up that occurred during MDBR operation. Song et al. [198] explored producing pure water and biogas using a coupled anaerobic membrane bioreactor–membrane distillation system. The authors concluded that the biogas production varied between 0.3 and 0.5 L/g COD added (about 65% methane) and that the conductivity of the distillate water was low. Nguyen et al. [196] confirmed that a hybrid system of attached growth biofilm and MDBR could reduce membrane fouling, remove nutrients, and produce drinkable water. However, the flux of the coupled system is not high. Therefore, further studies should research the optimization of overall system performance such as reactor design and the hydrodynamics of the system [41,199,200].

## 6. Conclusions and Future Outlook

This review first gives a brief overview of the fundamentals of MD and compares the advantages and disadvantages of different MD configurations. Then, it covers how MD is driven by waste heat and renewable energy sources and how MD is applied to treat concentrated solutions. Lastly, it describes the integration of MD with FO and the bioreactor process. The analysis of current MD research reveals that membrane distillation is an attractive desalination process due to its good capability to be integrated with other industrial processes such as solar energy, RO, MED, crystallization, FO, and bioreactors.

Despite the great potential of membrane distillation, its application and development have been hampered by the wetting and fouling of the membrane, low flux, and high energy consumption. Therefore, further studies related to membrane fouling control and scaling reduction are needed before the commercialization of MD systems for the disposal of concentrated brine. Simultaneously, studies on the modification of the membrane, novel membrane material, and the optimization of the membrane structure are needed to improve the anti-wetting properties of the membrane and to achieve high flux and a long-term stable process of concentrated brine treatment. An optimization of the overall system performance, such as the reactor design, the membrane module, and the hydrodynamics of the system, would be useful to increase energy efficiency and permeate flux. A detailed thermo-economic analysis would help its commercial implementation. Therefore, a heating technique, such as photothermal heating, electrothermal heating, or induction heating, coupled with MD is attractive because it directly heats the solution near the membrane surface, improving energy efficiency. In addition, further research on the coupling of membrane distillation with other systems is needed to expand its application.

## Figures and Tables

**Table 1:** List of recently published review papers cover the hybrid systems of MD process.

Author	Title	Year	Hybrid Systems	Ref.
Ullah et al.	Energy efficiency of direct contact membrane distillation	2018	Solar	[29]
Aamer et al.	Membrane technology in renewable-energy-driven desalination	2018	Solar and geothermal energy	[30]
Li et al.	Solar assisted sea water desalination: A review	2013	Solar	[31]
Saffarini et al.	Technical evaluation of stand-alone solar powered membrane distillation systems	2012	Solar	[32]
Charcosset	A view of membrane processes and renewable energies for desalination	2009	Solar	[33]
Pangarkar et al.	Review of membrane distillation process for water purification	2016	Solar and multi-effect MD	[34]
Wang et al.	Recent advances in membrane distillation processes: Membrane development, configuration design and application exploring	2015	Multi-effect MD, FO, crystallizer, bioreactor, and solar	[35]
Ashoor et al.	Principles and applications of direct contact membrane distillation (DCMD): A comprehensive review	2016	Solar, crystallization, waste heat, geothermal energy, freeze desalination, photocatalysis, UF, and FO	[19]
González et al.	Membrane distillation: Perspectives for sustainable and improved desalination	2017	Multi-effect MD, solar, waste heat, geothermal, and zero liquid discharge	[36]
Camacho et al.	Advances in Membrane Distillation for Water Desalination and Purification Applications	2013	RO, NF, FO, crystallization, solar, geothermal, and waste heat	[37]
Salmón et al.	Membrane crystallization via membrane distillation	2018	Crystallization	[38]
Jiang et al.	Progress in membrane distillation crystallization: Process models, crystallization control and innovative applications	2017	Crystallization	[39]
Bruggen	Integrated Membrane Separation Processes for Recycling of Valuable Wastewater Streams: Nanofiltration, Membrane Distillation, and Membrane Crystallizers Revisited	2013	NF and crystallizers	[40]
Goh et al.	Membrane Distillation Bioreactor (MDBR)—A lower Green-House-Gas (GHG) option for industrial wastewater reclamation	2015	Bioreactor	[41]
Zhang et al.	Review of thermal efficiency and heat recycling in membrane distillation processes	2015	Multi-effect MD	[42]
Curcio et al.	Membrane Distillation and Related Operations—A Review	2005	Crystallization	[16]
Gopi et al.	Perspective of renewable desalination by using membrane distillation		Solar	[43]
Ghaffour et al.	Membrane distillation hybrids for water production and energy efficiency enhancement: A critical review	2019	RO, BR, MVC, MED, MSF, MDC, AD, FO, and PRO	[44]
Choi et al.	Membrane distillation crystallization for brine mining and zero liquid discharge: opportunities, challenges, and recent progress	2019	Crystallization	[45]
Naidu et al.	Hybrid membrane distillation: Resource, nutrient and energy recovery	2020	Crystallizer, adsorbent, FO, bioreactor, PRO, and RED	[46]
Ahmed et al.	Alternative heating techniques in membrane distillation: A review	2020	Waste heat and solar	[47]

**Table 2:** Advantages and disadvantages analysis of conventional MD configurations.

Mode	Scheme	Advantages	Disadvantages
DCMD	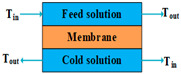	High permeability flux; Simplest configuration and minimum volume; High gained output ratio; Internal heat recovery is possible.	Highest conduction loss compared to other configurations; Low energy efficiency; Thermal polarization is high.
AGMD	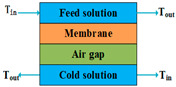	Internal heat recovery is possible; Lower conductive heat loss; Highest thermal efficiency; Less fouling tendency; Thermal polarization is low.	Permeate is easy to pollute; Mass transfer resistance is high; Lowest of permeate flux compared to others.
SGMD	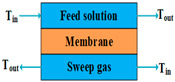	Low heat loss by conduction; Permeate flux is high.	Large footprint; Heat recovery is difficult; Pretreatment and control of sweep gas is intricate.
VMD	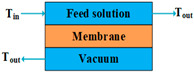	High permeate flux; Low heat loss by conduction.	Heat recovery is difficult; Membrane pore wetting is highly possible.
PGMD	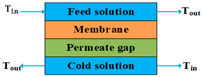	High permeate flux; Low heat loss by conduction.	Heat recovery is difficult; Permeate is easy to pollute.
OMD	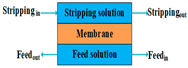	High permeate flux; Low heat loss by conduction; Concentrate solutions at lower temperature compared with other MD configurations.	Membrane pore wetting is highly possible; It is most often used to remove water from aqueous solutions; It is strongly affected by the concentration polarization.

**Table 3:** The performances of some solar-assisted MD systems.

Authors	Type of MD Module	Type of Study	Year	Result
Elzahaby et al. [48]	A tubular membrane for DCMD setup	Experiment and theory	2016	Maximum productivity: 40.587 kg/day Daily efficiency: 60.06% GOR: 0.624
Shim et al. [91]	Solar-powered DCMD	Experiment	2015	Heat energy consumption: 896 kW h/m^3^~1433 kW h/m^3^; GOR: 0.44~0.70
Kim et al. [51]	A solar-assisted hollow fiber DCMD module	Simulation	2013	Total distillate: 31,000 kg/day Specific thermal energy consumption: decreased
Chafidz et al. [76]	A solar-powered vacuum multi-effect membrane distillation system	Experiment	2016	Overall volume distillate: about 70 kg Conductivity: about 4.7 μS/cm Maximum production: 382.56 kg/day Flux: 1.5 L/(m^2^ h)~2.6 L/(m^2^ h)
Kabeel et al. [92]	Solar-driven DCMD	Experiment	2017	GOR: 0.49 Maximum productivity: 33.55 kg/day
Duong et al. [93]	A spiral-wound DCMD module	Simulation	2017	Distillate: 140 kg/day

**Table 4:** Summary the performance of some hybrid MD-PRO systems.

Draw Solution	Configuration	Nature of Work	Performance	Operating Conditions	Ref.
NaCl	PRO-MD	Simulation	Production of water and electricity. Energy efficiency: 9.8%.	Hot and cold working temperature: 60 °C and 20 °C.	[142]
NaCl	PRO-MD	Simulation	Production of water and electricity. Optimal thickness: ~90 µm. Pore radius: ~0.09 µm. Saving energy: 0.1738 kWh/m^3^. Loss of 3% permeate water flux.	Replacing the DCMD with the PRO-MD.	[143]
NaCl	PRO-MD	Experiment	Production of water and osmotic power. Ultrahigh power densities: 31 W/m^2^ and 9.3 W/m^2^.	Feed: deionized water and real wastewater	[140]
NaCl	MVDM-PRO	Simulation	Production of water and power generation. Maximum power density: 9.7 W/m^2^.	Feed: river water Flow rate: 0.5 kg/min	[147]
NaCl	RO-MD-PRO	Simulation	Production of water and energy generation. Outperforming stand-alone RO.	Specific energy consumption and the environmental footprint: reduced.	[144]
NaCl	RO-MD-PRO	Simulation	RO-MD-PRO has a higher energy efficiency than RO-PRO.	Using an economic heat source.	[146]

## Data Availability

No new data were created or analyzed in this study. Data sharing is not applicable to this article.
